# Which influenza viruses will emerge following the SARS‐CoV‐2 pandemic?

**DOI:** 10.1111/irv.12866

**Published:** 2021-05-06

**Authors:** Karen L. Laurie, Steve Rockman

**Affiliations:** ^1^ Seqirus Ltd Parkville Vic. Australia; ^2^ Department of Immunology and Microbiology The University of Melbourne Parkville Vic. Australia

**Keywords:** COVID‐19, influenza, pandemic, pandemic influenza, seasonal influenza, virus replacement

## Abstract

The world has experienced five pandemics in just over one hundred years, four due to influenza and one due to coronavirus (SARS‐CoV‐2). In each case of pandemic influenza, the pandemic influenza strain has replaced the previous seasonal influenza virus. Notably, throughout the SARS‐CoV‐2 pandemic, there has been a 99% reduction in influenza isolation globally. It is anticipated that influenza will re‐emerge following the SARS‐CoV‐2 pandemic and circulate again. The potential for which influenza viruses will emerge is examined.

## 1 INTRODUCTION

Four subtypes of influenza co‐circulate in the human population, causing epidemic infections. There are two influenza A subtypes, A(H1N1) and A(H3N2), and two influenza B lineages, B/Victoria/2/87 (B/Victoria) and B/Yamagata/16/88 (B/Yamagata), with variable domination each season. In the temperate Northern and Southern Hemispheres (NH/SH), a single peak incidence of infection is observed in the respective winter months. This seasonal variation of influenza in temperate areas leads to the specific timing of vaccination in an effort to minimise the burden of disease. Influenza activity in tropical and sub‐tropical countries is less well defined since these countries often detect influenza throughout the year with some having multiple peaks of influenza activity.^1^ The timing of epidemics is thought to be influenced by temperature, humidity, mode of transmission (droplet, fomite, direct contact) and human behaviours (eg indoor congregation).^1^

**FIGURE 1:**
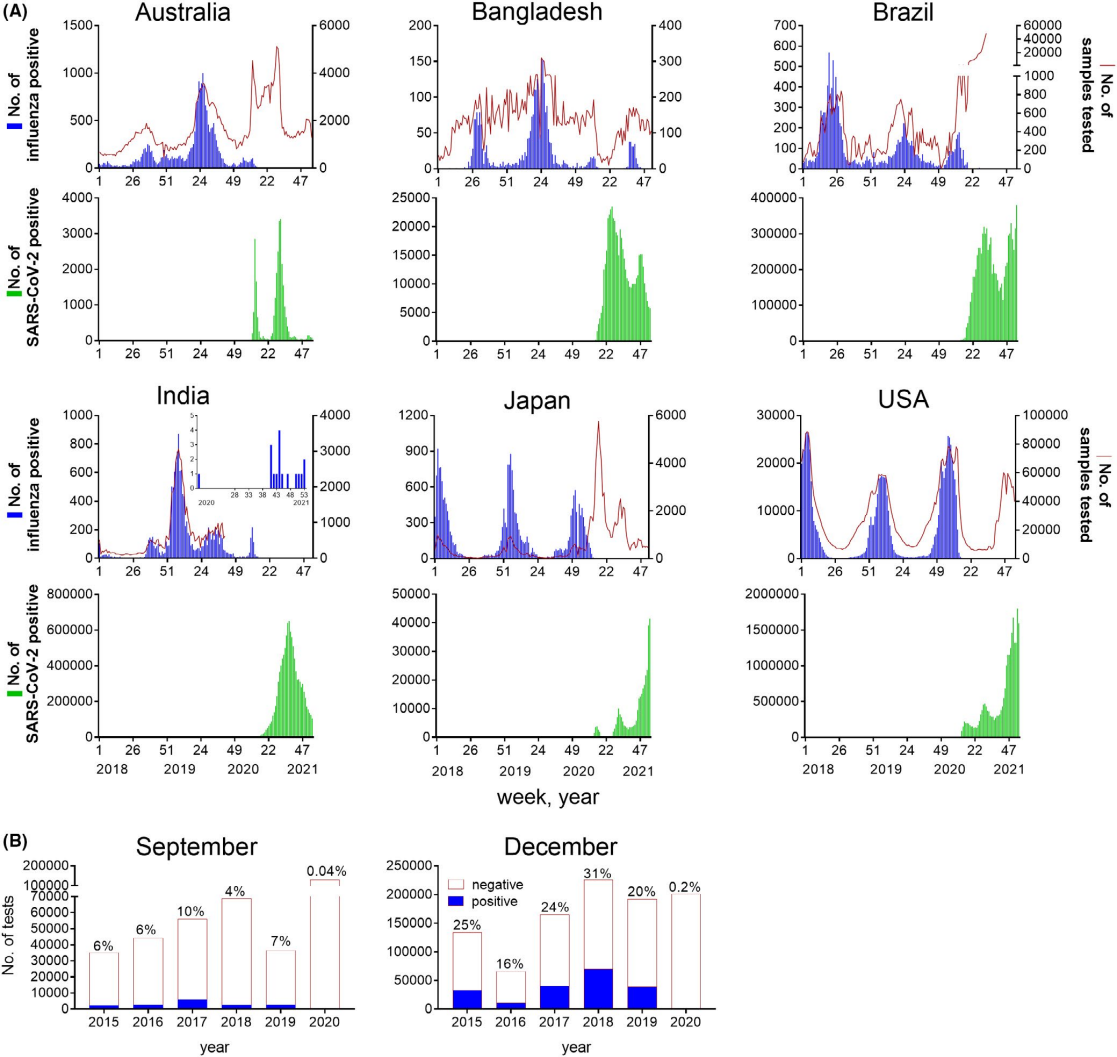
Epidemiology of influenza and SARS‐CoV‐2 infections. (**A**) Number of influenza positive samples (blue) and testing rate (red line) for influenza and number of SARS‐CoV‐2 positive samples (green) for countries indicated from week 1, 2018 to week 3, 2021. Influenza data collated from^4^ and SARS‐COV‐2 data collated from.^19^ Inset graph for India shows influenza positive samples from week 15, 2020 until week 3, 2021. (**B**) Number of influenza positive samples (blue) and negative samples (white) for influenza globally for first two weeks in September or last week of December/first week of January from 2015 to 2020/2021, as collated from.^5^ Percentage of positive tests indicated above each column. These time points were selected as representative of high detections of influenza virus circulating in Southern and Northern Hemispheres, respectively

In December 2019, the emergence of a novel coronavirus, SARS‐CoV‐2, was detected in Wuhan, China.^2^ Cases were rapidly detected in other countries as the virus spread, leading the World Health Organization (WHO) to declare a public health emergency of international concern (PHEIC) on 30 January 2020. As case numbers and deaths increased worldwide, the WHO declared the 2nd pandemic of the 21st century, on 11 March 2020.^2^ Multiple waves of infections have been observed in most countries since March/April 2020 as various containment measures have been repeatedly implemented and lifted (Figure 1A).

Strikingly, a dramatic decrease in influenza detection has been observed since March 2020 co‐incident with the exponential global spread of SARS‐CoV‐2, despite testing for influenza continuing at similar levels in many countries (Figure 1A).^3^, ^4^ Compared to 2015‐2019, the Global Influenza Surveillance and Response System (GISRS) reported a 99% reduction in the number of influenza positive cases in the first two weeks of September 2020 and the two weeks either side of the 2020/2021 new year (Figure 1B).^5^ Detections of other respiratory viruses (respiratory syncytial virus (RSV), parainfluenza) have also decreased in 2020.^6^, ^7^ Why have influenza detections drastically diminished during the SARS‐CoV‐2 pandemic? Will influenza return upon the introduction of mass vaccination for SARS‐CoV‐2? If so, which of the current influenza viruses will remerge, or will new influenza viruses emerge in their place?

## 2 EVIDENCE AND MECHANISMS OF RESPIRATORY VIRUS DOMINANCE

Non‐pharmaceutical mitigation strategies (social distancing, travel and movement restrictions, hygiene measures) are widely thought to have reduced influenza circulation.^3^ For example, in Australia and New Zealand the emerging 2020 SH influenza season was halted in March when mitigation strategies were strictly enforced to control COVID‐19 case numbers.^3^, ^4^

In locations where COVID‐19 is widespread, influenza is still significantly reduced. This relative absence of cases of human influenza virus infection throughout the SARS‐CoV‐2 pandemic has precedence. Historically, when a novel influenza virus has emerged and become pandemic, the circulating contemporary seasonal influenza virus strain has disappeared. For example, the pandemic A(H2N2) virus replaced the circulating A(H1N1) virus in 1957. The A(H2N2) virus became endemic until it was replaced with the novel A(H3N2) virus in 1968. The dominance of a novel virus (influenza or coronavirus) may occur when the global population is immunologically naïve to that virus (thus the vast majority of people are susceptible to infection with the new virus). This is the case for SARS‐CoV‐2, whereby the population is highly susceptible and the virus has increasing transmissibility.^8^ A “viral interference” or “temporary immunity,” whereby infection of one respiratory virus limits infection and replication of another virus, may also contribute. Viral interference has been observed for seasonal influenza/s and other respiratory viruses, such as RSV and rhinovirus.^9^, ^10^, ^11^ Mechanistically, it has been hypothesised that a localised inflammatory response, characterised by type I interferons, may be induced by the primary virus infection, rendering the respiratory tract refractory to another infection.^9^, ^11^

## 3 WHAT SHOULD WE EXPECT FOR INFLUENZA AS THE SARS‐CoV‐2 PANDEMIC SUBSIDES?

### 3.1 Sources of influenza and susceptibility—setting the scene for re‐emergence of influenza

Detection of occasional seasonal and pre‐pandemic influenza viruses in 2020 indicate influenza has not been eradicated.

Influenza A viruses circulating in avian and mammalian populations (such as swine) provide reservoirs for human infection.^1^ Sporadic zoonotic infections of influenza A virus occur in humans, reflecting the ability of these virus genomes to adapt to human receptor and hosts.^1^ Detection of zoonotic infections of influenza have continued in 2020 with similar (low) incidence as the past three years.^12^ Establishment of zoonotic influenza viruses to a human infection would likely follow the same steps to that of SARS‐CoV‐2. The initial emergence of SARS‐CoV‐2 resulted from the virus adapting to replicate in humans; close human‐to‐human contact (enhanced by domestic and international transport); a limited widespread use of hygiene practice and environmental conditions conducive to transmission such as temperature and humidity.^3^, ^13^

Intermittent cases of seasonal influenza detected since March 2020 demonstrate that suppression of influenza by SARS‐CoV‐2 disease is not absolute (Figure 1A).^4^, ^5^ A(H1N1), A(H3N2) and B/Victoria viruses have been isolated from Bangladesh, India, Cambodia, China and France in 2020, in extremely small numbers.^4^, ^5^ Notably, the majority of these influenza isolations are in tropical locations. Tropical locations of East and South East Asia and India have previously been identified as a global source of A(H3N2) activity (reviewed in^1^). There has been suggestion that the incidence of SARS‐CoV‐2 infections was lower in regions closer to the equator in the early months of the pandemic,^13^ which may have contributed to persistence of influenza in these areas. It is possible that this continuous low level virus circulation may allow for the seeding of influenza to the globe from tropical regions following significant commencement of international travel.

Immunity to influenza is thought to wane over time, though the longevity of antibodies and cellular memory response is dependent on a person's age, vaccination/infection history and time since their past exposure.^14^, ^15^ Although vaccination rates have been higher in 2020,^16^, ^17^ the lack of global circulation of influenza throughout 2020 and early 2021 may increase the number of those with reduced immunity to influenza through lack of exposure. Thus, there is potential for increased susceptibility of the population to infection with influenza, and severe disease. Influenza viruses continuously evolve to escape immune pressure in a process known as antigenic drift. Two drivers of antigenic drift are vaccination and natural infection, as both induce antibody responses primarily to the viral surface proteins, Haemagglutinin and Neuraminidase. A secondary effect of the reduction in population immunity is a potential for a limitation of antigenic drift as there is less immune pressure.

### 3.2 Potential scenarios for influenza

Following the roll out and widespread application of SARS‐CoV‐2 vaccines, increase in international travel and complacency around personal hygiene methods such as mask wearing and hand washing, it is inevitable that influenza will return. Four scenarios are possible;

(1) Seasonal influenza returns, and the same clades return as were circulating in late 2019/early 2020. Current vaccines would offer protection. However, influenza viruses isolated since April 2020 have been from limited genetic clades and subtypes compared to those circulating in 2019, suggesting that this scenario is unlikely.

(2) Seasonal influenza returns, yet some lineages disappear and there is reduced diversity in the clades of each subtype/lineage. As viruses from the B/Yamagata lineages have not been isolated in humans since early 2020, there is a significant possibility that the B/Yamagata lineage will not re‐emerge. This hypothesis is further supported by the cross reactivity of B/Victoria strains, and the lack of a zoonotic reservoir of B viruses.^1^, ^18^ Viruses that have persisted throughout 2020 have a fitness advantage that enabled their ongoing circulation (eg replication, immune escape). New quasi‐species or drifted variants may also emerge over time as more people become infected and viruses mutate to escape immune pressure (antigenic drift). Current vaccines may offer some protection.

(3) Influenza returns intermittently with occasional outbreaks. This scenario is unlikely due to the tropical reservoir of viruses that exist (see above), the eventual return of international travel and the modest efficacy of influenza vaccines.

(4) A new zoonotic influenza strain appears in the human population causing the next pandemic. Influenza continues to present a pandemic threat.

## 4 FUTURE

Along with uncertainty as to the re‐emergence of influenza virus strains, subtypes and lineages, there is also uncertainty as to the severity of future outbreaks given the “break” in epidemic influenza infections in 2020. The best strategy to protect humans going forward is to maintain high seasonal influenza vaccination rates. This is complemented by surveillance, monitoring and preparedness through GISRS.

It is inevitable that another respiratory human pandemic will occur but when, and via which vector (coronavirus, influenza, other), is unknown. While novel mRNA vaccines for COVID‐19 have been developed at unprecedented speed, the challenges of developing a four‐strain influenza vaccine with this technology should not be underestimated. We are fortunate that the global influenza system has led to the development of robust pandemic influenza preparedness, where egg, cell‐based, and recombinant technologies and adjuvants currently utilised for seasonal influenza vaccination can deliver safe and effective pandemic influenza vaccines across all age groups in a matter of months. The importance of understanding virus evolution, human susceptibility, ecological and social interactions are key to responding to the next pandemic.

## CONFLICT OF INTEREST

Karen Laurie and Steven Rockman are employees of Seqirus, an influenza vaccine manufacturer.

## AUTHOR CONTRIBUTIONS

**Karen L. Laurie:** Conceptualization (equal); Writing‐original draft (equal); Writing‐review & editing (equal). **Steven** **Rockman:** Conceptualization (equal); Writing‐original draft (equal); Writing‐review & editing (equal).

## Data Availability

The data were derived from the following resources available in the public domain: WHO. Influenza FluMart. 2021. https://apps.who.int/flumart/Default? ReportNo=12 and WHO. WHO Coronavirus Disease (COVID‐19) Dashboard. 2021. https://covid19.who.int/.
